# Sleep deprivation affects inflammatory marker expression in adipose tissue

**DOI:** 10.1186/1476-511X-9-125

**Published:** 2010-10-30

**Authors:** José C Rosa Neto, Fábio S Lira, Daniel P Venancio, Cláudio A Cunha, Lila M Oyama, Gustavo D Pimentel, Sérgio Tufik, Cláudia M Oller do Nascimento, Ronaldo VT Santos, Marco T de Mello

**Affiliations:** 1 Departmento de Fisiologia da Nutrição, Universidade Federal de São Paulo (UNIFESP)-São Paulo/SP, Brazil; 2 Departmento de Psicobiologia, Universidade Federal de São Paulo (UNIFESP) - São Paulo/SP, Brazil; 3 Associação de Suporte da Psicofarmacologia (AFIP), São Paulo/SP, Brazil; 4 Departmento de Biociências, Universidade Federal de São Paulo (UNIFESP), Baixada Santista Campus, São Paulo/SP, Brazil

## Abstract

Sleep deprivation has been shown to increase inflammatory markers in rat sera and peripheral blood mononuclear cells. Inflammation is a condition associated with pathologies such as obesity, cancer, and cardiovascular diseases. We investigated changes in the pro and anti-inflammatory cytokines and adipokines in different depots of white adipose tissue in rats. We also assessed lipid profiles and serum levels of corticosterone, leptin, and adiponectin after 96 hours of sleep deprivation.

**Methods:**

The study consisted of two groups: a control (C) group and a paradoxical sleep deprivation by 96 h (PSD) group. Ten rats were randomly assigned to either the control group (C) or the PSD. Mesenteric (MEAT) and retroperitoneal (RPAT) adipose tissue, liver and serum were collected following completion of the PSD protocol. Levels of interleukin (IL)-6, interleukin (IL)-10 and tumour necrosis factor (TNF)-α were analysed in MEAT and RPAT, and leptin, adiponectin, glucose, corticosterone and lipid profile levels were analysed in serum.

**Results:**

IL-6 levels were elevated in RPAT but remained unchanged in MEAT after PSD. IL-10 protein concentration was not altered in either depot, and TNF-α levels decreased in MEAT. Glucose, triglycerides (TG), VLDL and leptin decreased in serum after 96 hours of PSD; adiponectin was not altered and corticosterone was increased.

**Conclusion:**

PSD decreased fat mass and may modulate the cytokine content in different depots of adipose tissue. The inflammatory response was diminished in both depots of adipose tissue, with increased IL-6 levels in RPAT and decreased TNF-α protein concentrations in MEAT and increased levels of corticosterone in serum.

## Introduction

A large body of evidence has shown that prolonged paradoxical sleep deprivation (PSD) leads to a reduction in body mass, elevated energy metabolism, changes in circulating hormones, loss of immune integrity, and other disorders [1,2]. Moreover, sleep deprivation has produced hyperphagia, weight loss, increased energy expenditure, increased in plasma catecholamine concentration, hypothyroidism, reduction in core temperature, deterioration in physical appearance [3], and reduced levels of anabolic hormones [4]. Importantly, as the occurrence of sleep disturbances increases in modern lifestyles, so too does the risk of cardiovascular disease [5].

PSD induces an inflammatory response with increases in serum pro-inflammatory cytokines [6,7]. Seventy-two hours of PSD increased TNF-α, IL-6, IL-1α and IL-1β levels in the sera of rats [7]. C-reactive protein and IL-6 were increased in humans after sleep deprivation [8]. Additionally, a recent study showed that sleep restriction to 50% of the time habitually spent sleeping, over a 10 day period, induced significant increases in IL-6 levels in serum [9]. Chronic elevation of inflammatory proteins can contribute to health problems including cardiovascular, endocrine, mood, and sleep disorders [10]

Moreover, subjects with sleep loss showed an increase in nuclear factor NF-κB DNA binding in peripheral blood mononuclear cells [11]. NF-κB activation is thought to contribute to the pathophysiology of diseases such as Diabetes mellitus, cardiovascular disease, and atherosclerosis [12].

During situations that induce stress, like exhaustive exercise and cancer, adipose tissue shows increased expression of cytokines, which act both locally and distally and have autocrine, paracrine and endocrine effects [13,14]. The physiology, metabolism and function of white adipose tissue vary in a depot-specific manner [15]. Therefore, marked differences in gene expression amongst depots are reported for both rodents [16] and humans [17]; cytokine secretion is also heterogeneous. Adipose tissue is responsible for 30% of circulating IL-6 and TNF-α at rest [18], and in some diseases (e.g., obesity, Diabetes mellitus), this tissue contributes to systemic levels at even greater percentages [17].

The aim of this study was to examine the effects of PSD at 96 hours on the expression of pro-(TNF-α and IL-6) and anti-inflammatory (IL-10) cytokines in different depots of adipose tissue, as well as on the serum levels of the adipokines leptin and adiponectin.

## Methods

### Animals

Male *Wistar *rats, aged 3 months and weighing 300-350 g at the beginning of the experiment, were obtained from the Instituto Nacional de Farmacologia (INFAR) colony at UNIFESP. The rats were housed inside standard polypropylene cages in a temperature-controlled (23.1°C) room with a 12:12 h light-dark cycle (lights on at 06:00 hours). All procedures used in the present study complied according to the guide for care and use of laboratory animals, and the experimental protocol was approved by the UNIFESP Ethical Committee (0876/09).

### Paradoxical sleep deprivation procedure

The experimental groups were submitted to a single platform sleep deprivation method. The single platform-on-water (flower pot) method is extensively used for depriving paradoxical sleep. This technique involves placing the animal on a narrow circular platform (10 cm in height and 6.5 cm in diameter) placed inside a chamber (23 × 23 × 35 cm) filled with water to within 1 cm of their upper surface over a period of 96 h. At the onset of each paradoxical sleep episode, the animal experiences a loss of muscle tone and falls into the water, thus being awakened. The narrow platform procedure caused complete and selective loss of PS during all four days. Food and water were provided *ad libitum *by placing chow pellets and water bottles on a grid located on top of the chamber. The base of the feeder was adapted with a plate to prevent pieces of chow from falling into the water. The water in the chamber was changed daily throughout the PSD period. Control rats were placed inside the water chamber, but, instead of water, the chamber was filled with sawdust.

### Hormonal assay

After the PSD period, the rats were brought to an adjacent room and decapitated between 09:00 a.m. and 12:00 p.m. The control group rats were euthanised at the same time as the experimental group, by euthanised on the first day of the experimental procedure. Blood was collected in glass tubes and centrifuged to obtain samples of serum or plasma. Samples were maintained at -20°C until assay. Corticosterone concentrations were assayed by a double antibody RIA method designed specifically for rodents, using a commercial kit (ICN Biomedicals, Costa Mesa, CA, USA). The sensitivity of the assay is 0.25 ng dL^-1^. Serum leptin and adiponectin were quantified using specific enzyme immunoassays (Genese^®^, Brazil), following the manufacturer's instructions.

Following sacrifice, mesenteric (MEAT) and retroperitoneal (RPAT) white adipose tissue samples were removed, snap frozen in liquid nitrogen, and stored at -80°C.

### Lipid profile

Triglycerides (TG), HDL-cholesterol, and total cholesterol were assessed through commercial enzymatic kits (Labtest^®^, São Paulo, Brazil). VLDL and LDL-cholesterol as well as TG hepatic content were calculated according to previously reported methods [19,20]. Plasma glucose concentration was measured using the enzymatic colorimetric method (Biotécnica, São Paulo, Brazil).

### TNF-α, IL-10, IL-6, protein level determinations

Frozen tissues (0.1-0.3 g) were homogenised in RIPA buffer (0.625% Nonidet P-40, 0.625% sodium deoxycholate, 6.25 mM sodium phosphate, and 1 mM ethylene-diamine tetraacetic acid at pH 7.4) containing 10 μg/ml of a protease inhibitor cocktail (Sigma-Aldrich, St. Louis, Missouri). Homogenates were centrifuged at 12,000 × *g *for 10 min at 4°C, the supernatant was stored, and the protein concentration was determined using the Bradford assay (Bio-Rad, Hercules, California) with bovine serum albumin as a reference. Quantitative assessment of TNF-a, IL-6 and IL-10 was carried out by ELISA (DuoSet ELISA, R&D Systems, Minneapolis, MN). For the TNF-α (DY510), IL-6 (DY506) and IL-10 (DY522) assays, the sensitivity was found to be 5.0 pg/ml Intra- and inter-assay variability of the TNF-α and IL-6 kits was 2.7-5.2% and 4.9-9.5%, respectively. Assay sensitivity for IL-10 was 10 pg/ml. The intra-assay variability of the IL-10 kit was 2.0-4.2%, and its inter-assay variability was 3.3-6.4%. All samples were run as duplicates, and the mean values were reported.

### Statistical analysis

All data are expressed as mean ± standard deviation (SD). The Student's *t*-test was used to evaluate the statistical significance of differences between means. P-values of less than 0.05 were considered statistically significant.

## Results

Ten rats were randomly assigned to either the control group (C) (N = 5) or the 96 hour (PSD) group (N = 5). PSD treatment decreased weight of RPAT (-65.1%), MEAT (-68.3%) and liver (-35%). Fat content in liver tissue was decreased in the PSD group as compared to the C group (52.1%) (Table 1).

**Table 1:** Weight of RPAT, MEAT, liver and Fat content in liver.

Groups	Liver weight (g)	Hepatic TAG (mg TAG/100 mg liver)	MEAT weight (g)	RPAT weight (g)
**Control**	11.56 ± 1.47	22.03 ± 8.35	1.63 ± 0.50	4.91 ± 1.52
**PSD96h**	7.55 ± 0.69*	10.76 ± 6.28	0.57 ± 0.21*	1.72 ± 0.64*

Results are expressed as mean value ± SE. p < 0.05*, significantly different from control values.

### Metabolic profile

**Figure 1:**
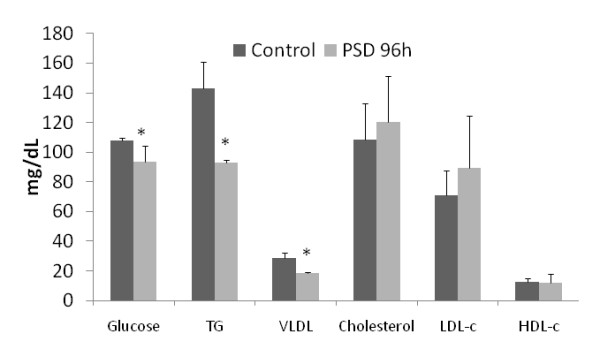
**Metabolic profile**.

Plasma levels of glucose (decrease 13.4% in the PSD group as compared to the control group), TG (-34.2%), and VLDL (-35.1%) were decreased in PSD. However, plasma levels of cholesterol, LDL cholesterol and HDL cholesterol did not differ between groups (Figure 1).

### Hormonal concentrations

**Figure 2:**
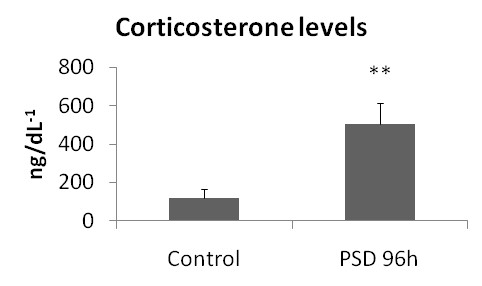
**Corticosterone level**.

Corticosterone concentrations increased at 96 h of PSD (+337.13%) with respect to the control group (Figure 2).

**Figure 3:**
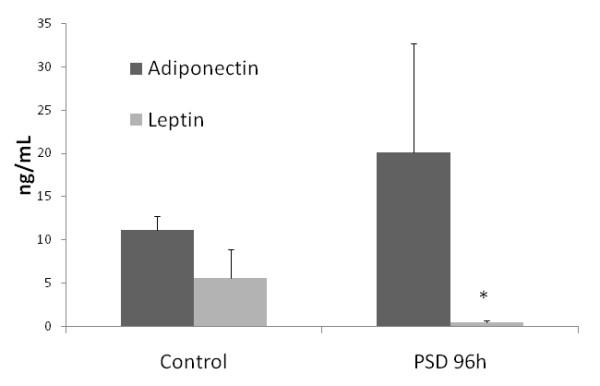
**Leptin and adiponectina levels**.

Leptin (-91,7%) was decreased and adiponectin plasma levels were not altered between groups (Figure 3).

### Cytokine concentration

**Figure 4:**
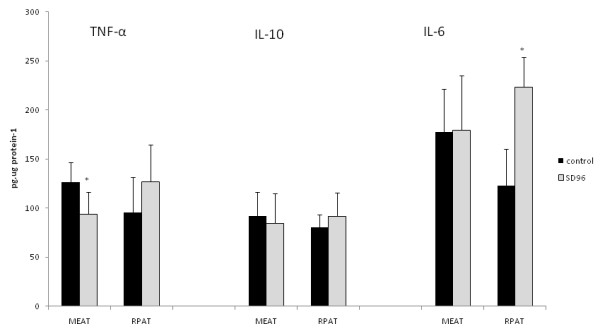
**Cytokines levels**.

IL-6 protein content was increased in RPAT (+81,3%), but not altered in MEAT after sleep deprivation (Figure 4). IL-10 protein content was not altered in either depot. TNF-α protein content was decreased in MEAT (-25%), but it was unchanged in RPAT in the PSD group as compared to the C group (Figure 4).

## Discussion

In this study, cytokines in adipose tissue were regulated after 96 hours of PSD in rats. In addition, the responses in different depots of adipose tissue were heterogeneous, as IL-6 levels increased in RPAT, but TNF-α concentrations decreased in MEAT. Moreover, lipid profiles were also altered by sleep deprivation. Corticosterone levels were greatly increased and leptin levels were dramatically decreased in serum after 96 hours of PSD.

A general reduction in the amount of time spent sleeping, as well as a greater frequency of more acute episodes of sleep deprivation, have become endemic conditions in modern society. This fact is highlighted by findings reported in the current literature, which identify important associations between sleep loss and alterations in nutritional aspects and metabolism [21-23]. Ninety-six hours of sleep deprivation in rats resulted in decreased plasma levels of glucose, TG and VLDL. This effect upon lipid metabolism is in agreement with a previous report [5].

Alterations in sleep duration influence control of food intake [24,25]. Many studies have demonstrated that sleep loss increases food intake, while decreasing body weight [1,3,26]. Increased food intake is associated with an imbalance in the anorexigenic hormone, leptin [27-29], and an increase in the orexigenic hormone ghrelin [29,30]. In our study, serum concentrations of leptin decreased dramatically after 96 hours of PSD sleep deprivation, associated with a reduction in adipose fat mass. Koban and Swinson [2] showed that leptin decreased after sleep deprivation and remained low following twenty days of recovery. Therefore, this factor may be associated with the drastic reduction in adipose tissue mass following sleep deprivation.

It is well established that PSD modulates levels of cytokines in the serum [7,10,31]. The effects on circulating levels of IL-6 after sleep deprivation are controversial. Studies have demonstrated that this cytokine can be elevated [6,32], decreased [10,33] or not measurably changed after sleep deprivation [31,34]. This conflict may in part be related to the dual pro- and anti-inflammatory roles of IL-6.

Our findings demonstrated that IL-6 was increased in RPAT but not altered in MEAT. This result can be explained by a heterogeneous difference in depots of adipose tissue; MEAT is more stable in the production of immune responses than RPAT [35]. In adipose tissue, IL-6 increases lipolysis, decreases lipase lipoprotein (LPL) activity and increases the mobilisation of fatty acids [36,37]. This increase in IL-6 in adipose tissue is similar to responses seen after exhaustive exercise [14], which increases metabolic demands similarly to PSD.

Sleep deprivation did not alter TNF-α in RPAT, but in MEAT this protein was decreased after 96 hours of sleep deprivation. This decrease in TNF-α can be associated with elevated systemic levels of corticosterone. The anti-inflammatory cytokine profile observed in different depots of adipose tissue agreed with a previous report [38] that showed increased IL-10 and decreased TNF-α expression in the brain after PSD. However, the systemic inflammatory profile after PSD is controversial. Studies have found that patients with sleep disturbance show altered circulating concentrations of TNF-α, IL-1β, and IL-1 [6,39]. However, other studies have failed to detect changes in circulating levels of IL-6 [31,34] and TNF-α [6] in relation to sleep deprivation. An important site of cytokine production includes monocytes. Lange et al. [39] showed that PSD influenced cells toward a Type 2 immune response. This abolishment of a Type 1 response and increase in Type 2 was associated with increased cortisol levels. Our results suggest that the increase in corticosterone levels up-regulates IL-6 but down-regulates TNF-α.

Sleep deprivation causes metabolic alterations with increases in energetic metabolism associated with anorexia, which can cause body weight loss. Therefore,, decreases in lipid serum, increases in lipolysis and decreases in adipose tissue depots are found in PSD rats.

In conclusion, adipose tissue is affected by sleep deprivation. Beyond loss of adipose tissue mass, sleep deprivation increases cytokine production primarily of anti-inflammatory molecules but also of lipolytic cytokines such as IL-6. In MEAT, TNF-α was decreased after sleep deprivation, demonstrating that PSD induces an inhibition in pro-inflammatory response in this tissue as well.

## Conflicts of interests

We declare that there are no conflicts of interest (including financial and other relationships) for each author.

## Authors' contributions

JCR, FSL, DPV, CAC, LMO, GDP, ST, CMON, RVTS and MTM participed the sample collected, assess samples, design of the study and performed the statistical analysis, and writing of paper. All authors read and approved the final manuscript
